# Advancing Maternal Health Through the EMBRACE Research Center of Excellence: Foundations, Approach and Protocol

**DOI:** 10.1111/1471-0528.18335

**Published:** 2025-08-26

**Authors:** Dara D. Méndez, Hyagriv N. Simhan, Sade A. Tukuru, Stacy Bartlett, Catherine L. Haggerty, Ashley Hill, Onome Oghifobibi, Cynthia Salter, Jada Shirriel, Ngozi Tibbs, Lovie J. J. Foster, Linda Adodoadji, Iris Ryn Olson, Mehret Birru Talabi

**Affiliations:** ^1^ Department of Epidemiology University of Pittsburgh School of Public Health Pittsburgh Pennsylvania USA; ^2^ Department of Obstetrics and Gynecology, Magee‐Women's Research Institute & Foundation, Magee‐Womens Research Institute204 Craft Avenue University of Pittsburgh School of Medicine Pittsburgh Pennsylvania USA; ^3^ Department of Family Medicine University of Pittsburgh School of Medicine Pittsburgh Pennsylvania USA; ^4^ Division of Community Health Sciences University of Illinois Chicago Chicago Illinois USA; ^5^ Division of Newborn Medicine, Rangos Research Center, Room 6135UPMC Children's Hospital of Pittsburgh UPMC Children's Hospital of Pittsburgh Pittsburgh Pennsylvania USA; ^6^ Department of Behavioral and Community Health Sciences University of Pittsburgh School of Public Health Pittsburgh Pennsylvania USA; ^7^ Healthy Start Inc. Pittsburgh Pennsylvania USA; ^8^ Journey Lighter Coaching Pittsburgh Pennsylvania USA; ^9^ Division of Rheumatology and Clinical Immunology University of Pittsburgh School of Medicine Pittsburgh Pennsylvania USA

**Keywords:** anti‐racism, birthing people, black maternal health, community centered research, health equity, postpartum care, reproductive justice, training

## Abstract

**Objective:**

To describe the development of the EMBRACE Center, which seeks to advance Black maternal health in Allegheny County through research and capacity building.

**Design:**

The EMBRACE Center is a multidisciplinary community‐academic research partnership at the University of Pittsburgh. Researchers and community collaborators—many of them Black‐led organisations—share power in ways that value diverse forms of knowledge.

**Setting:**

Allegheny County, Pennsylvania.

**Population or Sample:**

Birthing people in Allegheny County, Pennsylvania, and surrounding regions.

**Methods:**

The EMBRACE Center's Research Project seeks to enhance postpartum (4th Trimester) and interconception care that improves pregnancy outcomes and reduces rates of severe maternal morbidity among Black individuals. EMBRACE's community and training components facilitate community partnerships throughout research, implementation, training, and capacity building for reproductive justice and equity. Working groups focus on shared decision‐making, communication, and dissemination processes that enhance data collection and sharing with the community. A multi‐disciplinary advisory board and a community advisory board provide feedback to the Center.

**Main Outcome Measures:**

Research outcomes of interest include reducing maternal morbidity and mortality. Center outcome measures include development of a regional maternal health equity workforce.

**Results:**

Center activities include education in maternal health and reproductive justice, data justice community trainings, development of measures for structural and social determinants of maternal health, and convening community advocates, researchers and practitioners for black maternal health and reproductive justice.

**Conclusions:**

The work of EMBRACE will result in sustainable approaches to advance maternal and reproductive health equity and improve well‐being for black birthing People.

## 1 Introduction

Rates of severe maternal morbidity and maternal mortality in the United States are higher than those of comparably high‐income countries [1]. Up to one‐third of severe maternal morbidity events occur within the first 6 weeks after delivery [2], and the majority of maternal deaths occur during that first year. This underscores the significant vulnerability of birthing people—a term inclusive of women, transgender, and gender diverse individuals who are capable of pregnancy, during the postpartum period. Adverse maternal outcomes are particularly striking among historically marginalised populations, particularly Black birthing people. For example, the national incidence of maternal mortality among Black birthing people is 49.5 per 100 000 people, as compared to 19.0 to 100 000 among white birthing people [3]. A disproportionate burden of adverse maternal health is also observed regionally. For example, in Pennsylvania, Black people experience 14% of births but 23% of pregnancy‐associated deaths [4]. In Allegheny County, Pennsylvania, which includes the city of Pittsburgh, the inequality in maternal mortality rate between Black and white birthing people is higher than 84% of similar cities [5].

Racial inequities in birthing outcomes are rooted in historical oppression and structural racism that reinforce traditional power structures, discrimination, and racialized distribution of resources [6, 7]. This history manifests in Allegheny County [8, 9, 10, 11, 12] through inequitable [13, 14, 15, 16]: (1) access to quality clinical care and social services; (2) allocation of resources across communities; and (3) distribution of power and policies. Due to the complex intersections of structural and social determinants of health, Black birthing people also enter pregnancy with a disproportionate burden of chronic disease that contributes to adverse maternal health indices [17, 18].

While racial inequities in maternal health constitute an urgent public health crisis, outdated theories of change have undermined the development of effective and sustainable strategies to enhance Black maternal outcomes [19, 20]. As our formative research indicates, many initiatives are led by organisations that are not embedded in Black communities and take a “raise all boats” approach—focusing on equality rather than equity, which requires centering resources on individuals who have been disproportionately marginalised. Furthermore, some initiatives focused on Black maternal health lack the leadership and guidance of Black individuals.

This paper describes the development of Equity in Maternal and Birthing Outcomes and Reproductive Health through Community Engagement (EMBRACE), an academic‐community partnership housed at the University of Pittsburgh that was initiated in 2022. EMBRACE is also one of the Maternal Health Centers of Excellence across the United States, funded through the National Institutes of Health (NIH) Implementing a Maternal health and PRegnancy Outcomes Vision for Everyone (IMPROVE) Initiative [21]. This initiative currently includes 10 maternal health research centers as well as an Implementation Science Hub called AMETHIST, and a Data Innovation Hub. IMPROVE seeks to address social, biological, and structural factors associated with pregnancy‐related morbidity and mortality. EMBRACE seeks to advance reproductive health equity through multidisciplinary and community‐partnered research, training, clinical practice, and policy efforts in Allegheny County, Pennsylvania and surrounding regions.

## 2 Methods

### 2.1 Background and Center Origins

#### 2.1.1 Theoretical Frameworks

EMBRACE is guided by theoretical frameworks such as Reproductive Justice (RJ) and Public Health Critical Race Praxis. RJ, which was developed by a group of Black women in response to Clinton‐era health reform efforts in the 1990s, asserts that all people should be able to create, raise, and nurture the families that they desire, including having or choosing not to have a pregnancy, and parenting with dignity and necessary support systems [22]. Public Health Critical Race Praxis highlights how racism and social hierarchies operate on the health and well‐being of individuals and communities and emphasises the power structures that undergird racialized health inequities. PHCRP also asserts that knowledge generated from scientific research can be used to disrupt racial inequities and advance public health [23, 24].

To address health inequities that affect Black birthing people in Allegheny County, EMBRACE places primacy on the conduct of community‐partnered research that advances the health and wellness of Black birthing people; enhances resource acquisition for Black families; RJ and anti‐racism training for EMBRACE members; and collaborative research methods training and data justice, in which community members engage in formulating research agendas, implementing methods, analysing data and determining how research findings are disseminated.

Community partners include but are not limited to Healthy Start Pittsburgh [25], Journey Lighter Coaching [26], Yogamotif [27], and the Black Women's Policy Agenda [28] and EMBRACE continues to develop and nurture new community partnerships. Academic partners are situated at the University of Pittsburgh in the Schools of Public Health, Medicine, and Nursing, in addition to cross‐disciplinary university centers, including The Center for Health Equity, CONVERGE, Magee Women's Research Institute, and the University Center for Social and Urban Research. Healthcare partners include the University of Pittsburgh Medical Center, Allegheny Health Network, The Midwife Center, and the East Liberty Family Health Care Center. Public health partners include the Allegheny County Health Department. For clarity, we provide the abbreviations used throughout the paper (Table 1).

**TABLE 1:** List of abbreviations used.

Term	Meaning
EMBRACE	Equity in Maternal and Birthing Outcomes and Reproductive Health through Community Engagement
RJ	Reproductive justice
IMPROVE	Implementing a Maternal health and Pregnancy Outcomes Vision for Everyone
BEST	Advancing Birth Equity Strategies Together
BIRTH Plan	The Allegheny County Battling Inequities and Realising Transformational Health Outcomes Plan
MCH	Maternal and Child Health
4TM	4th Trimester Care—a model that enhances care in the postpartum period
ICC	Interconception Care—a model that enhances screening for risk factors between pregnancies
IMPLICIT	Interventions to Minimise Preterm and Low birthweight Infants through Continuous Improvement Techniques
CC	Community Component
TC	Training Component

#### 2.1.2 Center Development

Over the past decade, partners engaged in EMBRACE have led efforts to improve health outcomes of Black families through a number of aligned initiatives. The Advancing Birth Equity Strategies Together Allegheny Initiative [29], involving over 50 partners and organisations across Allegheny County [17], has enhanced the social, economic, and physical well‐being of communities through activities co‐led by Healthy Start Pittsburgh and the Allegheny County Health Department. Additionally, Healthy Start Pittsburgh led a strategic community‐informed process for the development of The Allegheny County Battling Inequities and Realising Transformational Health Outcomes (BIRTH) Plan for Black Babies and Families [30]. The BIRTH Plan includes four key areas: (1) Strengthen the maternal and child health (MCH) Workforce; (2) Strengthen Systems of Care; (3) Address Social Determinants of Health; and (4) Coordinate and Streamline MCH Initiatives. The BIRTH Plan, ongoing research at academic centers such as the University of Pittsburgh, and preceding community initiatives informed the selection of EMBRACE's key projects and components (Table 2). The IMPROVE initiative launched in 2019 and presented a funding opportunity to further enhance the maternal health goals of the region more broadly and EMBRACE specifically, by bringing together community, academic, and government partners with a long history of work in addressing maternal and child health outcomes in Allegheny County. EMBRACE‐specific initiatives were guided by collective decision‐making at the outset during the conceptualization phase.

**TABLE 2:** The extension and grounding of Allegheny County BIRTH plan priorities into EMBRACE center of excellence projects and activities.

Action areas	EMBRACE center project or component	Description^a^
1. Strengthen the MCH workforce^a^	4TM/ICC project Training Component (TC) Community Component (CC)	4TM/ICC: Development of Community Health Workers (CHWs) and clinicians responsible for addressing project aims within health systems TC: Strengthens the maternal health workforce through training and capacity building
2. Strengthen systems of care^a^	4TM/ICC project	Strengthens processes for equitable interconception and 4th trimester care interventions across multiple sites along with major community healthcare partners across the region
3. Address social determinants of health^a^	Proposed projects 4TM/ICC project	Proposed research will focus on structural and social determinants of health 4TM/ICC: Piloting an unconditional cash transfer for birthing people
4. Coordinate and streamline MCH initiatives	Overall EMBRACE	EMBRACE Center goals and activities align with BIRTH plan priority areas, including: “Assess and Strengthen MCH Organisations' and Collaboratives' Equity” and “Increase equitable funding for MCH initiatives in the region”

^a^ The Community Component of EMBRACE includes community‐led processes for research, training, intervention, and dissemination in maternal and reproductive health through all projects and components. The BIRTH plan was co‐developed by the Allegheny County MCH Strategy team.

EMBRACE will continue to coordinate and streamline MCH initiatives across Allegheny County by fostering collaboration between organisations and funders working in this field.

#### 2.1.3 Center Structure

**FIGURE 1:**
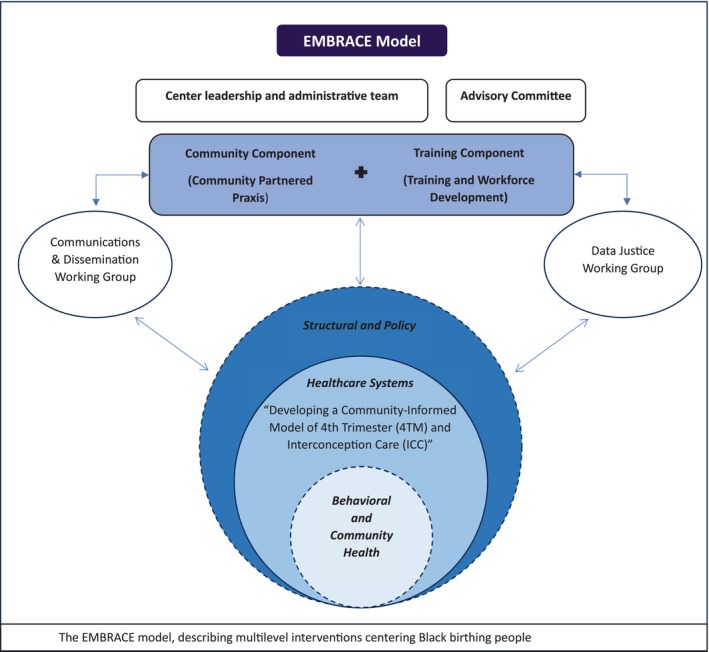
The EMBRACE model.

EMBRACE takes a multi‐level approach to understanding and promoting optimal maternal health using a modified social ecological model (See Figure 1) [31] through research related to community health, equitable healthcare systems, and interventions at the structural and policy level; particularly how these interventions impact Black birthing people. The key research project is situated at the health systems level. This project entails integrating racial equity and RJ into the clinical care delivery of postpartum and interconception care for Black birthing people via the Interventions to Minimise Preterm and Low birthweight Infants through Continuous Improvement Techniques (IMPLICIT) network. Two additional intervention studies are in development and further explicate our multi‐level approach: a community‐based health worker model to address perinatal co‐morbidities (behavioural level), and a policy intervention study (policy level) investigating the implications of new state and federal maternal health policies on maternal morbidity and mortality (these projects were not funded under the NIH U54 and are in pilot phases) (Figure 1). The research projects are supported by the Community Component (CC), which includes community leaders and academic partners who provide expertise and insight into multiple aspects of EMBRACE. EMBRACE's Training Component (TC) provides education and training in MCH equity, RJ, anti‐racism, and trauma‐informed care. Each project within the EMBRACE Center is led by an academic and community‐based researcher who serve as multiple Principal Investigators (MPIs). This leadership structure allows for a balance of responsibilities, perspectives, and decision‐making, facilitating deep and comprehensive connections with community members and voices rather than including community partners only as ad hoc consultants. EMBRACE includes numerous Black researchers and Black‐led organisations as Co‐PIs, consultants, or advisors. Each research project and the Community and Training Components include at least one underrepresented early‐stage investigator who will receive mentorship and support to lead innovative community‐partnered research. This approach is intended to nurture a new cadre of underrepresented investigators who are deeply informed in RJ and community participatory research and will have the tools to sustainably advance maternal health equity among Black populations. All EMBRACE staff, trainees, investigators, and collaborators will participate in training in racial consciousness, anti‐oppression, and RJ. Ongoing learning and professional development opportunities will reinforce and expand the EMBRACE community's knowledge and practice in these domains.

## 3 Results

### 3.1 Research Project: Developing a Community‐Informed Model of Fourth Trimester and Interconception Care (4TM/ICC) (Domain: Healthcare Institutions and Systems)

In Pittsburgh, Pennsylvania, Black people's pregnancy‐related mortality is higher than nearly 90% of similar cities [5]. Most severe maternal morbidity and maternal mortality events occur in the postpartum period—in many cases, once people have returned home after delivery. Most existing postpartum care models include a single maternal health visit between 6 and 8 weeks after delivery. However, 40% of birthing people do not attend their postpartum clinic visits [32], and Black women are 3.5 times more likely to miss postpartum visits than white women [33]. In part, these data reflect structural realities; one in four women return to work within 10 days of delivery [34], particularly individuals whose employers do not provide paid maternity leave. Individuals with chronic medical conditions or pregnancy‐associated conditions (e.g., gestational hypertension and diabetes) may therefore not receive the follow‐up needed to mitigate risk factors for severe maternal morbidity and maternal mortality. These gaps in postpartum healthcare delivery may help to explain the abysmal rates of adverse maternal health outcomes in this group.

However, while many people miss their postpartum clinic visits, over 90% of postpartum individuals attend their infants' well‐child visits [35]. This has informed the development of clinical care models to address risk factors for adverse maternal health outcomes during well‐child visits.

One nationally recognised model is the IMPLICIT Network, a 15‐year consortium of 38 U.S. family medicine and paediatrics practices that use quality improvement to enhance prenatal and interconception care (ICC). The current ICC model screens mothers for smoking, depression, multivitamin use, and family planning at well‐child visits, but does not provide clinical care or physical examinations. The IMPLICIT Network's Pittsburgh sites, situated in the University of Pittsburgh Medical Center Family Medicine Department, have also piloted 4th Trimester Care, which includes early identification of risk factors for postpartum complications, an enhanced discharge process, nurse phone check‐in at 1 week, an early postpartum visit within 3 weeks, and a traditional comprehensive postpartum visit at 6–8 weeks.

Interconception and 4th Trimester Care are increasingly being recognised as key strategies for preventing severe maternal morbidity and maternal mortality [36]. While the existing IMPLICIT ICC and 4TM projects have been piloted as quality improvement initiatives, a lack of funding, in part, has precluded the rigorous evaluation of maternal and infant outcomes from these models of care, or the standardisation of approaches at Pittsburgh's various IMPLICIT sites. The current IMPLICIT model does not focus on care delivery for individuals at highest risk of adverse maternal outcomes such as Black birthing people, and does not yet address racialized inequities in healthcare (e.g., provider bias, coercion, and discriminatory attitudes and actions that might lead birthing people to disengage with the healthcare system).

Drawing on the strengths of the current IMPLICIT model, including the longevity of the intervention and preexisting implementation data, the EMBRACE Center will develop a new postpartum care model, building on the ICC and 4TM interventions. Anti‐racism and anti‐oppression training will be provided for clinicians and patient‐interfacing staff members to highlight the positive health outcomes that can arise from person‐centered care that is autonomy‐supportive and respectful. Research approaches will also include focus groups with community members, patients in the current IMPLICIT network, and clinicians and staff involved in the IMPLICIT network. The team will also analyse clinical outcomes of patients in the current IMPLICIT network and in the larger obstetric population in Allegheny County, which will be used as a comparison for clinical outcomes in the new clinical care model.

EMBRACE will also pilot test an unconditional cash transfer that mirrors paid maternity leave who are simultaneously receiving ICC/4TM care. The goal is to target existing structural inequities, particularly with policy implications, and to assess whether enhanced financial resources improve maternal and infant clinic visit attendance, adherence to treatment recommendations and follow‐up care, and long‐term outcomes such as improved mental health, reduced rates of postpartum depression, and enhanced nutrition and maternal wellness (Table 3).

**TABLE 3:** Progress toward Allegheny County BIRTH plan priorities via EMBRACE centre of excellence projects and activities.

Action areas	EMBRACE center project or component	Description
1. Strengthen the MCH workforce	4TM/ICC project Training Component (TC) Community Component (CC)	4TM/ICC & CC: – Inclusion of financially supported Community Health Advocates in the EMBRACE model TC: – Internship program for graduate students – Mentorship program for post‐doctoral and early‐career faculty – Supporting maternal and child health faculty from underrepresented backgrounds – Provision of reproductive justice training
2. Strengthen systems of care	4TM/ICC project	– Development of focus group guides for clinicians and patients to collect data about barriers and facilitators of postpartum care
3. Address social determinants of health	Proposed projects 4TM/ICC project	Proposed projects: Seeking partners to continue research development and conducting pilot studies 4TM/ICC: Further development of the cash transfer pilot study and program Further development of a database related to measures of structural and social determinants of health to be applied across multiple studies and interventions
4. Coordinate and streamline MCH initiatives	Overall EMBRACE	EMBRACE serves as an academic hub for maternal and child health in Allegheny County by: – Connecting synergistic initiatives and organisations – Encouraging collaboration between existing organisations and projects – Building a coalition for collective action to promote Black maternal and reproductive health equity

The IMPLICIT ICC/4TM research project will yield actionable strategies for identifying and implementing health supports for Black people in the early postpartum period to prevent maternal morbidity and advance health equity in postpartum care in primary care settings.

### 3.2 Community Component

The EMBRACE Center Community Component provides community expertise, support, and oversight over all EMBRACE activities. The CC is comprised of trusted community leaders, organisations and institutions. It supports individuals and agencies in building community, capacity, and infrastructure for advancing Black maternal health and eliminating racialized maternal health disparities. The CC engages in team science to provide guidance and ensure community integration for all proposed research, training, and data collection, and to develop a collaborative network of maternal health agencies that promote the expertise of community advocates by drawing from the strengths and accomplishments within the region. Community partners are embedded in all aspects of the research lifecycle—from question generation to implementation and dissemination of research projects, scholarship, training, and capacity [37, 38, 39]. We apply the Diffusion of Innovation approach, which asserts that there are different stages in the innovation process and that individuals move through these stages at different rates and with different concerns [40]. This approach will be implemented while centering the concept of Cultural Humility, which emphasises lifelong learning and critical self‐reflection, power imbalances and institutional accountability, while we pursue systems change [41]. The CC includes partnerships across multiple sectors committed to improving the birthing and family wellness landscape in Allegheny County and leverages a robust network of existing community‐engaged research initiatives, intervening at the community level of the social ecological model.

Activities associated with the CC include training Community Health Advocates to be involved with EMBRACE components and teams, co‐development of research projects with community partners, dispensing of funds for community‐led activities, and support of early investigators in community‐partnered research praxis. For example, Community Health Advocates from EMBRACE community partner, Healthy Start Inc., are involved in the 4TM/ICC research project—they participated along with academic researchers, in qualitative research and data analysis methods training, and provide community expertise on proposed research methods (Table 3). Community engagement will be evaluated through surveys, focus groups, and attendance‐tallying to collect and incorporate feedback about community needs, contributions, and mutual benefit. The vision of the CC is to create a collaborative network of maternal health agencies in the region that promotes the expertise of community advocates and advance maternal health care and outcomes.

### 3.3 Training Component

The Training Component facilitates bidirectional training for all EMBRACE members and collaborators to elevate their consciousness around racial and reproductive justice. This component offers training for workers and community members across the maternal health equity research and care continuum, engaging stakeholders at both the community and health systems levels of the EMBRACE social ecological model. The Training Component is guided by four aims: mentorship development, research enrichment, community integration and reproductive health justice. One TC goal is to promote the success of early‐stage community‐engaged reproductive and perinatal health equity investigators across multiple disciplines and levels, including early career faculty, postdoctoral and predoctoral scholars, and college and high school students. The TC also provides capacity building and training related to community‐engaged research, RJ, anti‐racism, anti‐oppression and humanity‐centered care. Ultimately, the TC supports a community‐centered and culturally responsive workforce of clinicians, researchers, and community and organisational partners.

The TC will advance a maternal health equity workforce trained in anti‐oppression and RJ frameworks. Activities include internships for graduate students and trainees in maternal and child health equity, pilot grant supports for early career investigators, MCH training for medical students, a seminar series focused on MCH and RJ experts with special focus on Black scholarship and community leadership, training a new cohort of Community Health Advocates, and ongoing educational opportunities around trauma‐informed care, obstetric racism, community‐based participatory research and public health praxis (Table 3).

### 3.4 Additional Components of the EMBRACE Center

EMBRACE has additional working groups to support ongoing activities related to communications and data justice and transparency. The EMBRACE Communication and Dissemination working group relays research findings to the public, lawmakers, the academic community, and community‐based organisations through seminars and public events, digital channels, and news outlets. This group has also developed innovative methods to communicate with wider audiences about research findings, including community forums and arts‐based dissemination. For example, the EMBRACE Center features an artist‐in‐residence, Alecia Dawn Young, founder of Yogamotif, who has expertise in postpartum yoga and the creative arts and is co‐developing EMBRACE initiatives focused on Black maternal wellness. The Data Justice working group supports research projects through data access, engagement in data‐related training activities, and translation and dissemination of research findings to the communities served by EMBRACE. This group also coordinates the monitoring and evaluation plans that were developed by each component of EMBRACE, in addition to a center‐wide monitoring and evaluation plan that will guide the assessment of progress toward goals. All EMBRACE investigators and partners receive training in data justice and literacy to ensure transparency of processes with the communities that are served by EMBRACE.

## 4 Discussion

### 4.1 Main Findings

EMBRACE aims to implement innovative approaches to advance maternal health equity and justice. This includes integrating RJ and Public Health Critical Race Praxis in shaping the research conducted and the interventions implemented. EMBRACE also applies a community‐informed vision for enhancing Black maternal health in Allegheny County—the BIRTH Plan—which was developed through years of collaboration between Centre leaders and partners. This serves as an innovative strategy for priority‐setting and the foundation for EMBRACE's work. The EMBRACE model is innovative in many ways and has set the standard for what maternal and reproductive health research can and should look like. Through a multi‐faceted approach that includes community engagement, rigorous science, education, and comprehensive training, EMBRACE will improve health outcomes among Black birthing people throughout the region. Birthing people will benefit from increased connectivity between MCH and reproductive health‐focused organisations, using EMBRACE as the hub for obtaining information and resources. In addition, community voices will be amplified, as EMBRACE seeks and incorporates their lived experiences into each aspect of its activities. Through dissemination of training opportunities throughout the career pipeline for a diverse range of MCH and reproductive health professionals, EMBRACE will also support the workforce and economic needs of the region. By engaging both community health workers and clinicians, EMBRACE is positioned to educate and support a well‐informed healthcare system that values the contributions of the social determinants of health to one's well‐being. EMBRACE also seeks to build sustainable relationships for its activities to have a lasting impact, helping to change the trajectory of health outcomes for birthing people for years to come (Table 3).

### 4.2 Strengths and Limitations

A current limitation for EMBRACE includes gaps in strategic partnerships that would enhance the impact of the Center. Building partnerships with governmental entities in addition to the local health department and a small group of dedicated lawmakers working on MCH, initiating new partnerships with community‐based MCH/RJ groups, and garnering philanthropic support would widen EMBRACE's impact. EMBRACE aims to foster community‐led research and practice; however, there is a need to further disseminate information across academic and community channels. EMBRACE is continuing to expand its network and continually iterating through processes to improve communication between the various partners. A notable strength of the EMBRACE model is its focus on bi‐directional participation and learning between academic and community collaborators. Each component of the model is led by multiple Principal Investigators—one from an academic setting and one from a community setting. This ensures that the community voice is fundamentally integrated into all decisions and processes. Another strength is the deliberate inclusion and mentorship of Black and underrepresented researchers, and local Black‐led organisations, helping to tailor research and intervention efforts toward the needs of this specific community.

### 4.3 Interpretation

There is a growing understanding in public health that addressing our nation's maternal and reproductive health crisis requires an interdisciplinary approach that centres on equity frameworks. Despite the grave consequences of neglecting the needs of birthing people, this area of research has been historically under‐funded and thus has many opportunities for growth [42]. Recent studies continue to highlight the disparities in healthcare and the many structural barriers that Black birthing people face, especially in the perinatal period [43]. To address this need, it is crucial to utilise innovative, multi‐level community‐collaborative approaches such as what EMBRACE has created and is iteratively implementing. The current research activities of EMBRACE have wide‐reaching implications for the future of postpartum care. The result of the 4TM/ICC project will be a new model of postpartum and interconception care that can be implemented as a new standard of care, improving outcomes in this vulnerable period. Together with other Maternal and Child Health Research Centres of Excellence, EMBRACE is synergising the MCH landscape and can serve as a model for research centres across the nation.

## 5 Conclusion

This protocol paper details EMBRACE as a model of collective, multi‐disciplinary action research praxis to advance reproductive justice and maternal health equity for Black birthing people. The work of EMBRACE will provide important insights into the strengths, needs and goals of Black birthing people in our communities, informing future research and intervention efforts. The foundation of community and equity‐centered expertise will also support the development of structural policies that will codify maternal health promotion for Black birthing people in the region and beyond.

## Author Contributions

Dara D. Méndez, Hyagriv N. Simhan, Stacy Bartlett, Catherine L. Haggerty, Ashley Hill, Onome Oghifobibi, Cynthia Salter, Jada Shirriel, Ngozi Tibbs, Iris Ryn Olson, and Mehret Birru Talabi contributed significantly to the conception and design of EMBRACE. Dara D. Méndez, Hyagriv N. Simhan, Stacy Bartlett, Catherine L. Haggerty, Ashley Hill, Onome Oghifobibi, Cynthia Salter, Jada Shirriel, Ngozi Tibbs, Lovie J. J. Foster, Iris Ryn Olson, Mehret Birru Talabi, Sade A. Tukuru, and Linda Adodoadji contributed to drafting and revising the article's thematic content and flow as well as contributed to the acquisition and analysis of data. Dara D. Méndez, Hyagriv N. Simhan, Stacy Bartlett, Catherine L. Haggerty, Ashley Hill, Onome Oghifobibi, Cynthia Salter, Jada Shirriel, Ngozi Tibbs, Lovie J. J. Foster, Iris Ryn Olson, Mehret Birru Talabi, Sade A. Tukuru, and Linda Adodoadji all gave final approval of the manuscript and agreeagreed to take responsibility for all aspects of the work and addressing all questions.

## Ethics Statement

The research study described in this paper has been approved by the University of Pittsburgh Institutional Review Board.

## Conflicts of Interest

The authors declare no conflicts of interest.

## Data Availability

The data that support the findings of this study are available from the corresponding author upon reasonable request.
